# Biometrics for Internet-of-Things Security: A Review

**DOI:** 10.3390/s21186163

**Published:** 2021-09-14

**Authors:** Wencheng Yang, Song Wang, Nor Masri Sahri, Nickson M. Karie, Mohiuddin Ahmed, Craig Valli

**Affiliations:** 1 Security Research Institute, School of Science, Edith Cowan University, Cyber Security Cooperative Research Centre, Joondalup, WA 6027, Australia; masri.sahri@gmail.com (N.M.S.); n.karie@ecu.edu.au (N.M.K.); mohiuddin.ahmed@ecu.edu.au (M.A.); c.valli@ecu.edu.au (C.V.); 2 School of Engineering and Mathematical Sciences, La Trobe University, Melbourne, VIC 3086, Australia; song.wang@latrobe.edu.au

**Keywords:** biometrics, IoT, security, access control, authentication, encryption

## Abstract

The large number of Internet-of-Things (IoT) devices that need interaction between smart devices and consumers makes security critical to an IoT environment. Biometrics offers an interesting window of opportunity to improve the usability and security of IoT and can play a significant role in securing a wide range of emerging IoT devices to address security challenges. The purpose of this review is to provide a comprehensive survey on the current biometrics research in IoT security, especially focusing on two important aspects, authentication and encryption. Regarding authentication, contemporary biometric-based authentication systems for IoT are discussed and classified based on different biometric traits and the number of biometric traits employed in the system. As for encryption, biometric-cryptographic systems, which integrate biometrics with cryptography and take advantage of both to provide enhanced security for IoT, are thoroughly reviewed and discussed. Moreover, challenges arising from applying biometrics to IoT and potential solutions are identified and analyzed. With an insight into the state-of-the-art research in biometrics for IoT security, this review paper helps advance the study in the field and assists researchers in gaining a good understanding of forward-looking issues and future research directions.

## 1. Introduction

**Figure 1:**
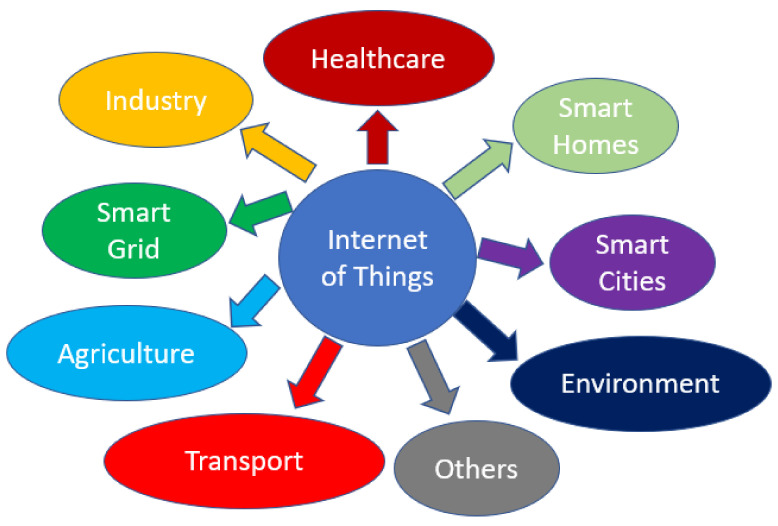
Application domains of the IoT (adapted from [7]).

The Internet of Things (IoT) contains a variety of devices, such as wearable devices, smartphones, computers, personal digital assistants (PDAs), and tablets. These devices, which consist of embedded sensors and processors that can handle their internal states or the external environment around them have become part of people’s daily necessities because of their decreasing cost, mobility and increasing computational capability. IoT includes a great diversity of smart devices collaborating to bring convenience and accessibility to people’s lives [1]. The benefits of IoT are immense and its applications are revolutionizing the manner in which we work and live. It also generates new opportunities for innovation, growth and knowledge sharing between different entities [2]. With a sharp increase in the number of IoT devices, these interconnected smart devices can be deployed in a variety of fields and their applications include but are not limited to smart homes, smart cities, environment, agriculture, smart grid, industry, healthcare, and transport. Application domains of the IoT are illustrated in Figure 1.

However, the low power and limited computing capacity constraints do not allow sophisticated security policies on IoT devices. The large number of interconnected IoT devices provokes a rapid increase in attacks from adversaries. With far inadequate awareness of IoT device users and vendors on the perils of IoT security, these IoT devices, in turn, are becoming a source of potential risks. Attackers can gain control of certain internal and open environments by accessing and probing into IoT devices (e.g., water outages, shortage of public electronic supply and tampering with the functionality of devices). Such security threats are concerning [3]; a house attached to any IoT device is an open invitation to attackers. In light of the above-mentioned security risks for IoT devices, it is vital to have proper access control in order to protect user privacy and prevent on-device data from being leaked [4]. Passwords used to be the only way of user authentication in the IoT, but times have changed. In the past decade, biometric technology has developed in leaps and bounds and has swiftly spread to almost every corner of our daily lives as a more reliable method of authentication. With the popularity of smartphones, it is a winning combination of mobile phones and biometrics in the consumer market, allowing biometric authentication to be more widely accepted. Since Apple started its incredibly usable biometric recognition, named Touch ID, in 2013 [5], the application of biometrics has expanded rapidly. Currently, IoT devices mounted with biometric modules are standalone products in the market. A recent biometric technology market data report published by ABI Research forecast that biometric recognition systems in smartphones would grow significantly to make a 95 percent penetration rate of smartphone shipments by 2022 [6].

Biometric recognition uses physical traits of human beings (e.g., fingerprint and face) for identification or verification. It overcomes the drawback of password-based authentication [8] and is becoming more prevalent with advancements in sensing technologies. Although biometric systems are advantageous over traditional password-based authentication, biometric information stored as template data in central databases or smart devices is vulnerable because any individual’s biometric traits cannot be changed or reissued, similar to passwords. Once biometric template data are compromised, the effect is forever, and privacy infringement and security breaches are likely to occur. If an adversary gains access to IoT devices using stolen biometric templates, it can put sensitive data or records stored in the devices at serious risk. Therefore, the importance of protecting biometric template data is twofold. First, user identity should be protected such that original biometric information cannot be retrieved. Second, users’ private data or sensitive information stored in IoT devices should only be accessed by genuine users.

A number of research articles integrate biometrics into IoT systems to secure IoT devices and applications. Ren et al. [9] surveyed the technologies and challenges of applying biometrics to the IoT. Moreover, the authors provided their visions on using biometrics in areas such as big data and mobile cloud computing. Subha [10] presented the advantages and disadvantages brought to the IoT by biometrics. Distinctive features of biometrics in relation to IoT security are summarized in [10]. Blasco et al. [11] conducted a survey on biometrics for wearable IoT devices. The authors discussed the differences between biometrics for wearable devices (called wearable biometrics in [11]) and traditional biometrics. In addition, the computing cost, system structure and experiment setup of different schemes are compared and analyzed in [11]. Obaidat et al. [12] discussed and compared popular biometric traits applied to the IoT in terms of accuracy, ease of use and cost. The authors also covered the biometric feature extraction techniques (e.g., the Fourier transform and Gabor filtering) and gave an example of a biometric-based eHealth system.

While the above-mentioned research articles provided insights into incorporating biometrics in IoT applications, to the best of our knowledge, little comprehensive survey over biometrics for IoT security has been conducted thus far, and in particular, the topics such as biometric data protection and biometric-cryptography in the IoT are not covered. To fill this gap, this paper presents an in-depth review of current research in biometrics for IoT security, especially focusing on authentication and encryption. The main contributions of this paper are highlighted as follows.

1. To the best of our knowledge, there has been little survey which adequately considers biometric authentication and encryption simultaneously for the IoT environment prior to this review paper. This paper gives a comprehensive review of the contemporary biometric-based systems that provide authentication and encryption for the IoT;
2. Regarding authentication, we classify and analyze IoT-related biometric authentication systems based on different biometric traits and the number of biometric traits used in the biometric systems. Following this, we investigate biometric-cryptographic systems that integrate biometrics with cryptography for data encryption. The study of these systems sheds light on the latest development in handling IoT-related security vulnerabilities or possible attacks targeting the IoT;
3. Challenges brought by the deployment of biometric systems in the IoT are identified and potential solutions are discussed and highlighted;
4. Several insights into future research directions concerning biometrics for IoT security are provided in this paper.

The database search strategy using keywords is adopted in this review paper such that a more complete list of articles can be obtained. In search of research papers, a number of databases (e.g., IEEE Xplore, Web of Science, Science Direct and Scopus) are included, followed by applying the inclusion and exclusion criteria based on titles and abstracts to filter out irrelevant articles. Then, the full text of each article is examined to determine whether the article should be included in this review paper, according to certain guidelines (e.g., [13]).

The remainder of the paper is organized as follows. Section 2 presents challenges and vulnerabilities identified in the IoT, while Section 3 describes common biometric traits that can be applied to the IoT. Section 4 discusses and classifies existing biometric-based systems that are being used purposely for IoT authentication. In Section 5, biometric systems that are applied to IoT data encryption are analyzed and discussed. Challenges brought by applying biometrics to IoT are highlighted in Section 6. Potential solutions and opportunities are presented in Section 7. Threats to the validity of this survey are discussed in Section 8. Section 9 concludes the paper and provides future research directions.

## 2. Security Challenges and Vulnerabilities of the IoT

Due to the limitations and constraints of the IoT in terms of computing capability, power and ubiquity, many security challenges are present in the IoT. The wireless technology, scalability, energy and distributed nature of the IoT are several of the main causes of security challenges [14]. According to [15], there are several underlying factors of the security challenges in the IoT:

(i) The weakest parts of a system. As the number of IoT devices is rapidly growing, the resource limitations of IoT devices lead to the use of lightweight security algorithms and the security of certain devices is likely neglected. These devices become the weakest parts of an IoT network;
(ii) Low control over updates. Often, users have a shallow understanding of the internal mechanisms of IoT devices and little knowledge about how to handle online updates, opening up opportunities for security attacks by various malware;
(iii) Data privacy. Smart sensors in IoT networks collect large amounts of data from different sources, and a certain amount of data may be related to users’ personal and sensitive information. The leakage of these data endangers the privacy of users.

The security challenges in the IoT can be overcome by authentication, confidentiality, integrity, and end-to-end security. Security principles, such as confidentiality, integrity, availability, authentication, lightweight solutions, key management systems and policies, should be implemented to enable a secure communication architecture [14]. Due to the variety of devices and communication protocols in an IoT setting as well as the diverse interfaces and services available in IoT, it is inappropriate to implement security mitigation based on conventional IT network solutions. Security measures (e.g., authentication through cryptographically pre-shared keys) currently utilized in traditional networks may not be adequate [16]. There are many attacks targeting IoT systems of a typical three-layer architecture, which is composed of the perception layer, network layer and application layer [17]. Vulnerabilities of or possible attacks to each layer [2] and security requirements in each layer [18] are listed in Table 1 and expounded in this section.

### 2.1. Vulnerabilities of the Perception Layer

The perception layer is responsible for sensing and gathering information about the surrounding environment through sensors. It can perceive certain physical parameters in the environment or identify other smart objects in the environment. Attacks on this layer mainly target the IoT nodes and are designed to compromise user privacy and reveal sensitive personal information [19]. These attacks include node tampering, radio frequency (RF) interference, node jamming, malicious node injection, physical damage and malicious code injection.

### 2.2. Vulnerabilities of the Network Layer

The network layer is responsible for connecting smart objects, network devices and servers. This layer is also used to transmit and process sensor data. Network attacks aim to manipulate the IoT network to cause damage and these attacks include traffic analysis attacks, radio-frequency identification (RFID) spoofing and cloning, man-in-the-middle attacks, routing information attacks, denial of service and Sybil attacks.

### 2.3. Vulnerabilities of the Application Layer

The application layer oversees application-specific services to users. It specifies the variety of applications for which IoT can deploy, for example, smart homes, smart cities and smart health. Application layer attacks include phishing attacks, viruses, worms, trojan horses and denial of service.

There are separate solutions for each attack listed in Table 1, however, if we have all these solutions implemented in the IoT, it will produce significant overheads to the operation of IoT and degrade its performance [2]. Among these security measures, authentication and encryption are the core requirements in defending possible attacks to each layer. Therefore, biometric-based systems designed for authentication and encryption to enhance IoT security are reviewed and analyzed in the following sections.

## 3. Biometrics Overview

Biometrics is a technology of identifying or verifying people based on their physiological and behavioral characteristics or traits. The selection of biometric traits should follow requirements such as universality, distinctiveness, permanence, and collectability. In addition, requirements for recognition accuracy and matching speed are to be met in the design of a practical biometric system. In Section 3.1, we classify and introduce common biometric traits, while in Section 3.2, we present performance metrics of biometric authentication systems.

### 3.1. Classification of Biometrics

**Figure 2:**
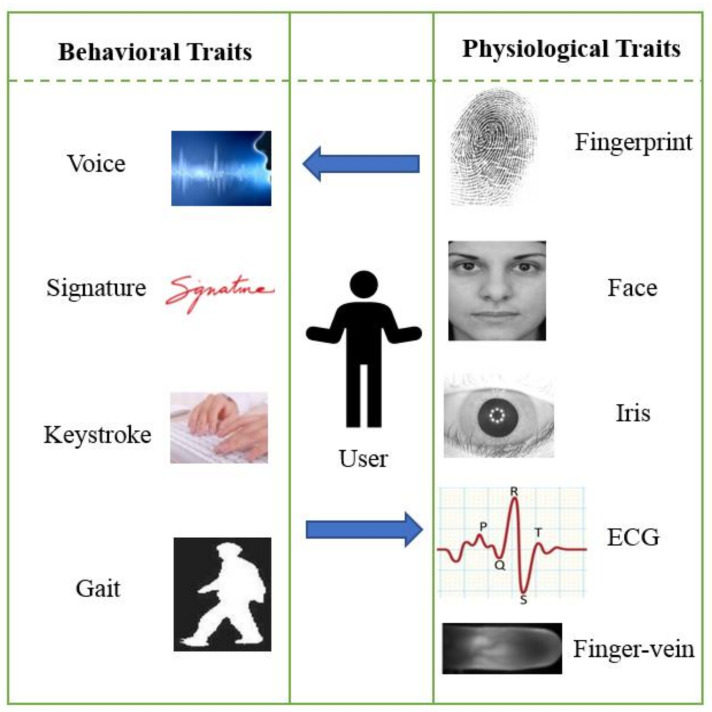
Examples of common biometric traits that can be used in authentication systems for IoT (adapted from [20]).

Several biometric traits that meet these requirements are generally classified into two categories, physiological traits and behavioral traits, as shown in Figure 2. Each biometric trait has its strengths and drawbacks; thus, the selection of biometric traits for authentication and encryption purposes depends on specific IoT applications [12]. Below is a brief introduction to common biometric traits.

#### 3.1.1. Fingerprint

A fingerprint is a mark left by the friction ridges of an individual’s fingertip. Fingerprints have been used in personal identification applications for centuries due to their convenience and high recognition accuracy [21]. The fingerprint pattern of ridges and valleys located on the fingertip surface is determined in the early stage of fetal development. Different persons’ fingerprints are different, including if they are identical twins [21]. Fingerprints are highly preferred because of their high recognition accuracy and user acceptability [22].

#### 3.1.2. Face

The face is the front part of the head of a person from the forehead to the chin, between which the mouth, nose, cheeks and eyes are included. Face recognition uses the spatial geometry of distinguishing features from the face image and is a topic of visual pattern recognition. Here, a face, as a three-dimensional object affected by different illumination, pose, expression, etc., is to be recognized based on its image. Face recognition has become increasingly popular due to the rapid development in the areas of smart cameras and mobile devices and demands for security and convenience [23]. A practical face recognition system is expected to recognize faces presented in images and videos in the model of verification or identification [23].

#### 3.1.3. Electrocardiogram (ECG)

The ECG is a signal recording of the electrical activity of the heart. Electrodes positioned on a person’s body surface are utilized to measure the electrical signals from the heart muscle. The ECG consists of three main components: P wave, QRS complex and T wave, where P, Q, R, S and T are special points defined in the ECG signal as shown in Figure 2. The P wave happens as a result of atrial depolarization, the QRS complex is caused by ventricular depolarization and the T wave occurs because of ventricular repolarization. There are three key characteristics associated with the use of ECG. First, ECG signals are difficult to falsify under supervised conditions. Second, ECG signals are present only in living individuals [24]. Third, ECG signals provide additional information related to psychological states, physiological and clinical states [24]. Table 2 summarized biometric authentication systems.

#### 3.1.4. Voice

The voice of an individual combines both behavioral and physiological elements. The shape and size of vocal tracts and nasal cavities are the physiological elements, while the movements of the jaw, lip and tongue are the behavioral elements. In voice recognition, duration, pitch information, intensity and quality of a vocal sound are the spectral information that is usually used to verify a user’s identity [12].

#### 3.1.5. Others

In addition to the common biometric traits introduced above, there are other traits, such as iris, palmprint, finger-vein, signature and keystroke.

*Iris*: It is a thin, annular structure in the eye. The variabilities of iris patterns between different individuals are enormous. Iris has a great mathematical advantage compared with other biometric traits. Besides, the iris is an internal organ of the eye, making it less likely to be affected by the environment and thus staying stable over time [49].

*Palmprint*: It includes flexion creases and distinct ridges similar to a fingerprint. Minutiae and creases are common feature representations that are extracted for authentication purposes [50].

*Finger-vein*: It is the pattern of blood vessels concealed under the finger skin and is distinct among individuals, including among identical twins [51].

*Signature*: This is a behavioral biometric trait often used in our daily business transactions. In signature recognition, the pressure, acceleration, speed and other attributes can be captured as features used in the matching process [52].

*Keystroke*: It is the typing pattern of an individual. Because of its high intraclass variability, recognition based on keystrokes faces huge challenges [53].

### 3.2. Evaluation of Biometric Authentication

Due to physiological, behavioral and environment factors in the biometric acquisition process, biometric uncertainty and noise in biometric authentication systems are inevitable, such as elastic distortion in fingerprint images [54]. It is most likely that samples from the same biometric trait captured at different times or under different conditions are different. Such variabilities may lead to authentication failure in a genuine attempt or fake success in an imposter attempt [55]. Performance of a biometric authentication system can be evaluated using a number of metrics, such as false acceptance rate (FAR), false rejection rate (FRR), equal error rate (EER) and recognition accuracy (RA). These performance measurement metrics are also applicable to biometric authentication systems in the IoT. The description of these metrics is as follows.

- False acceptance rate (FAR): The FAR is the probability of mistaking biometric samples from different subjects to be from the same subject [56].
- False rejection rate (FRR): The FRR is the probability of mistaking biometric samples from the same subject to be from different subjects [56].
- Equal error rate (EER): The EER is the error rate when FAR and FRR have the same value [56]. The FAR and FRR are inversely related, which means that when one increases, the other should decrease.
- Recognition accuracy (RA): RA is computed as the percentage of correct predictions out of the total number of observations. This metric is a common performance measure in machine and deep learning-based schemes [57].

## 4. Biometric-Based Systems for IoT-Oriented Authentication

There are several issues related to the sensitive data sensed and transferred by IoT devices. Transmission of data gathered by IoT devices to a remote server is likely to prompt security threats such as interception, interruption, modification, and fabrication. These threats can compromise user privacy, data integrity, confidentiality and service availability [58]. Authentication plays an essential role in establishing trust among users, IoT devices and IoT services and is considered a key solution to security issues in the IoT. Authentication ensures that received data come from correct devices and users and transmitted data are sent to intended recipients [58]. If an IoT device is accessed by an illegitimate user, the authentication mechanism should be able to detect it at any point during the surveillance session [58]. A well-functioning authentication system not only guarantees security in the IoT but also improves system trustworthiness [58]. Weak authentication can incur many attacks such as denial of service attacks and man-in-the-middle attacks [59].

**Figure 3:**
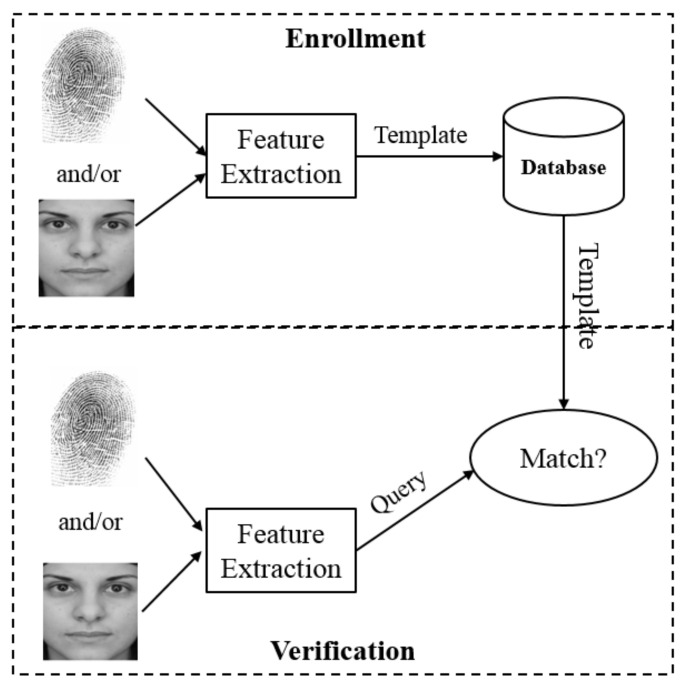
An example of a typical biometric authentication system (adapted from [22]).

A typical biometric authentication system is demonstrated in Figure 3. Biometric authentication is composed of two phases, namely the enrollment phase and the verification phase. In the enrollment phase, a set of features are extracted from the user’s biometric image (e.g., fingerprint image and/or face image) and stored in a central database or on a smartcard as template data. In the verification phase, the query’s biometric features are extracted in the same way as the enrollment phase and then compared against the template data in the matching module. If the similarity score between the template data and query data is larger than a predefined threshold, the verification is successful; otherwise, it is unsuccessful. Based on the number of biometric traits employed, biometric authentication systems can be categorized into single- and multi-modal biometric authentication systems, which are summarized in Table 2 and reviewed in Section 4.1. In Section 4.2, we analyze and discuss existing biometric authentication and key agreement schemes.

### 4.1. Single-Modal Versus Multi-Modal Biometric Authentication Systems

#### 4.1.1. Single-Modal Biometric Authentication Systems

A single-modal biometric authentication system uses information from only one biometric trait (e.g., fingerprint or face) for user authentication to prevent unauthorized access to IoT devices and services. A biometric-based authentication system offers convenience and strong security compared to conventional password-based authentication. There is ongoing, intensive research in biometric authentication systems for the IoT [9]. The incorporation of biometrics in the IoT is also called the internet of biometric things (IoBT), first introduced by Kantarci et al. [60]. Single-modal biometric authentication systems using different traits that appear in existing articles are reviewed below.

*Fingerprint*: Devikar et al. [25] proposed an attendance system applying fingerprint-based biometric authentication on a portable IoT device. Moreover, the cloud is used to store the attendance records, making data easy to be accessed and retrieved. Shah and Bharadi [26] introduced how to build a low-cost biometric system using Raspberry Pi, which is similar to a credit-sized mini-computer. In this study, the Raspberry Pi is used as a remote node and the enrolled biometric information (e.g., fingerprint data) is encrypted by a cryptographic algorithm and stored in the cloud. Prakash and Venkatram [27] enhanced home-IoT security using biometric authentication. In this paper, a fingerprint-based authentication system is implemented with Raspberry Pi together with several types of sensors. Taheri and Yuan [28] developed a cross-layer biometric recognition system that includes both software and hardware layers in a chaining structure. In this work, biometrics (e.g., fingerprint) is adopted. Sarika et al. [29] proposed a door lock system using fingerprints to unlock doors. The proposed system is composed of a fingerprint scanner, a magnetic solenoid lock, an Arduino board, and an LCD. This system is considered a good replacement of keys, locks, and cards and brings convenience and efficiency to organizations such as banks. Golec et al. [31] presented a biometric scheme named BioSec to provide user authentication for edge devices in IoT and Industry 4.0. Specifically, fingerprint-based biometrics are utilized in BioSec, and fingerprint data is secured in both the transmission channel and the database using the standard symmetric encryption method.

*Face*: With more and more IoT devices embedded with camera sensors in numerous applications in different industries, Hossain et al. [32] proposed a framework for biometric-based end-to-end IoT authentication as a security solution and included face recognition as a case study. Thilagavathi and Suthendran [33] conducted automatic real-time face recognition from videos using existing algorithms such as Adaboost and local binary pattern histograms. The Haar features extracted from the face images are used for face authentication. Gayathri et al. [34] integrated biometric authentication into the green IoT to secure the personal assistants (e.g., Google Assistant, Alexa and Cortana). In this system, local binary pattern histograms as face features are extracted and integrated with machine learning to achieve user authentication in the green IoT scenario. Kolhar et al. [35] proposed a decentralized IoT authentication framework based on face recognition in the background of lockdowns during COVID-19 outbreaks. The proposed edge computing framework includes three layers, namely physical layer, edge layer and face detection using the convolutional neural network (CNN). The proposed scheme is evaluated on several benchmark databases (e.g., WIDER FACE) and the performance in terms of recognition accuracy and latency is compared with state-of-the-art face recognition methods.

*ECG*: Karimian et al. [36] developed an ECG-based biometric system that acts as the communication interface between users and IoT devices for authentication purposes. The ECG is utilized in this study given its advantages in security, convenience, and implementation over other biometric traits. Hussein et al. [37] presented a real-time ECG-based authentication system for IoT. The discrete cosine transform (DCT) is used to extract the ECG feature vectors. This system can achieve an accuracy rate of 97.78% with a processing time of 1.21 s. Karimian et al. [61] implemented a biometric system making use of techniques such as obfuscation and physically unclonable functions to mitigate the risk of compromising biometrics under different malicious attacks. Moreover, a noise-aware biometric quantization framework is proposed in this work to generate unique, reliable keys with reduced processing time. Without complex processing, Barros et al. [38] proposed a feature selection algorithm utilizing fiducial points from the ECG signal records. The number of features used is less than 10, achieving 98.2% recognition accuracy over the stress recognition database. Karimian et al. [39] proposed a biometric quantization framework that can generate unique and high-entropy keys costing less enrollment time. The experimental results show that extracting the key from ECG rather than other biometric traits makes a good trade-off between reliability, security, and cost.

*Voice*: Shin and Jun [40] applied a voice-based recognition system to increasing the security and convenience of the home-IoT devices as it is a security risk if the home-IoT devices are operated by unauthorized voices. By recognizing the identity and analyzing the commands of the user, the voice recognition system ensures that only authorized users can control the home-IoT devices. Duraibi et al. [41] investigated the suitability of using voice for IoT authentication. In this research, the techniques and tools applied to voice authentication are reviewed and discussed. There are two phases of the proposed system, the enrollment phase and the verification phase. Specifically, in the enrollment phase, noise is first removed from the voice signals and then feature extraction is conducted. After that, the extracted features are input into a machine learning algorithm for training. In the verification phase, the query voice features are extracted in the same way as in the enrollment phase and tested against the trained model for verification.

*Others*: Habib et al. [58] presented an authentication framework for the IoT in eHealth using biometric modules and wireless device fingerprinting. The proposed framework verifies whether the sensed data comes from the correct patient and ascertains the integrity of the data received. The patient’s behavioral features are used for continuous biometric authentication. When the received biometric data cannot match the stored template, the system assesses whether the patient suffers a heart attack and if so, an alarm is triggered at the server and a notification is issued to the hospital system. In comparison with password-based or two-factor authentication methods, Lu et al. [42] explored finger-vein-based user authentication to achieve a more secure IoT environment. In the proposed scheme, an efficient local descriptor, called histogram of competitive orientations and magnitudes, is used to represent the finger-vein patterns in finger-vein impressions. Gad et al. [43] designed an iris recognition system to secure the communication between the IoT devices and the broker server. The two feature vectors extracted by different algorithms are fused into one. The size of the fused feature vector is then reduced and input into the Euclidian Distance classifier for classification. Meena et al. [62] presented a conceptual view about the application of iris-based authentication in the IoT. The authors identify the difficulty of applying iris authentication to IoT devices. In this paper, the scale-invariant feature transform is used for feature extraction and an artificial neural network-based classification algorithm is employed for predicting the user’s class.

In this sub-section, a large number of biometric-based authentication systems involving a single biometric trait are overviewed. Among them, the fingerprint is the most frequently used biometric trait due to its convenience and high accuracy offered by fingerprint recognition, while the ECG is most popular in the IoT healthcare field.

#### 4.1.2. Multi-Modal Biometric Authentication Systems

Multi-modal biometrics refers to the use of multiple sources of biometric information. Multi-modal biometric systems combine biometric evidence from multiple biometric traits (e.g., face and fingerprint) to improve recognition accuracy [63]. The obvious advantages of a multi-modal biometric system over its single-modal counterpart include better recognition accuracy and stronger security [64,65]. Regarding recognition accuracy, multi-modal biometric systems collect and fuse data from more than one trait. The fused data are more discriminative, enabling multi-modal biometric systems to perform better than single-modal biometric systems [64]. In terms of security, multi-modal biometric systems are more robust [65]. If one modal fails for an unknown reason, then another modal can still work to obtain authentication. Moreover, the use of multi-modal biometric systems increases the difficulty for attackers to spoof multiple biometric traits of an individual [65].

With more and more IoT devices equipped with high-resolution cameras, Macek et al. [44] presumed that it is possible to capture iris and face images simultaneously with a multi-modal biometric system. In this paper, the biometric features extracted from the captured iris and face images with fiducial point localization and Gabor filtering are stored on the IoT devices as templates. In the authentication stage, the stored templates are used to identify and authenticate IoT device users. Shahim et al. [45] designed a biometric authentication system for information security and access control of IoT devices. The proposed system uses both hand geometry scan and gesture of the user for authentication purposes. Machine learning techniques are also applied to feature classification.

Olazabal et al. [46] proposed a face- and voice-based multi-modal biometric scheme for resource-limited IoT devices. The proposed scheme fuses feature data from face images and voice signals using discriminant correlation analysis (DCA) and classifies the features through the K-nearest neighbors (KNN) algorithm. Benefitted from the feature fusion, the multi-modal system increases recognition accuracy by more than 50% compared to that using only face or voice features. To strengthen network security, Hassen et al. [47] extracted a private key from multi-modal biometrics (fingerprint and finger-vein) to authenticate and validate blockchain transactions. Experiment results demonstrate that the proposed method attains a high security level in defending spoofing and signature forgery with high throughput and low latency. Cherifi et al. [48] utilized ear and arm gesture features for user authentication in the application scenario where users answer phone calls. In this work, similarity scores are computed according to ear features using local phase quantization and arm gesture features based on four statistical metrics. Furthermore, a score-level fusion mechanism is used to calculate the final matching score such that the overall system performance is enhanced.

In this sub-section, several multi-modal biometric systems used for IoT-oriented authentication are reviewed. It follows that combinations of biometric traits may vary, for example, iris and face, face and voice, fingerprint and finger-vein, or ear and arm gesture features. However, no one combination is absolutely better than the others and the selection of combinations is application dependent. With pros and cons, it is not always desirable to use multi-modal biometrics. It remains an open issue as to how to effectively combine/fuse multiple biometric features and it is unclear about the exact cost of applying more than one biometric trait. None of these topics are discussed in any of the above papers.

#### 4.1.3. Comparison of Single-Modal and Multi-Modal Biometric Authentication Systems

As far as real-world implementations are concerned, single-modal biometric authentication systems that operate on a single biometric trait have issues such as noise in the sensed data, intraclass variation and interclass similarity, which can affect recognition accuracy. In contrast, multi-modal biometric authentication systems often outperform their single-modal counterparts by fusing multiple sources of information through different fusion strategies, such as feature-level fusion, score-level fusion and decision-level fusion [66].

Table 2 reports the performance in terms of EER or RA of many existing single-modal and multi-modal biometric authentication systems. Specifically, it can be seen from Table 2 that biometric traits with more discriminative features (e.g., iris) tend to achieve superior performance. For example, the EER of 0.2% for iris authentication [43] is better than the EER of 0.36% for finger-vein authentication [42]. In addition, multi-modal biometric authentication systems perform better than unimodal systems. For instance, in [46] the EER is 8.04% with fused data from face and voice, in contrast to the EER of 14.05% with features from only face and the EER of 43.76% with features from only voice.

Despite the advantages in recognition accuracy offered by multi-modal biometrics, issues pertinent to IoT applications should be considered. For example, stronger user cooperation is required for a multi-modal biometric authentication system, which may lead to inconvenience to IoT users. Moreover, collection, storage, and processing of multiple sources of information increase the complexity of the overall system and consume more resources. Given that most IoT devices have limited resources (e.g., storage, computational capability, bandwidth and power) [67], the benefits and drawbacks of implementing a multi-modal biometric authentication system in the IoT should be given full consideration.

It can be seen from Table 2 that the common hardware for testing the proposed single-modal or multi-modal biometric-based methods is Raspberry Pi, and about half of the methods are tested by using simulators instead of real IoT devices. Certain methods listed in Table 2 only describe how to implement the basic biometric authentication function on IoT devices (e.g., Raspberry Pi) but with no information about recognition accuracy or security; thus, no recognition performance of these methods can be included in Table 2. It also manifests that applying biometrics to the IoT is not yet popular or mature. There are various reasons for this, among which the main reasons may be the constraints of IoT devices (e.g., limited memory and computing power) and user acceptability.

#### 4.1.4. Continuous Biometric Authentication

Most of the biometric systems discussed above are generally referred to as static authentication systems. In a static authentication system, a user’s identity is authenticated at the beginning of a session, for instance, by logging in the IoT device using a fingerprint or entering the room using an iris scan. As opposed to static authentication, there is continuous authentication. In a continuous authentication case, the authenticity of the user’s identity is checked throughout the entire logon period.

Bours [68] proposed a method for evaluating a continuous keystroke dynamics system. Not only are the keystroke dynamics used at the beginning of a session, but they are continuously checked to monitor the user’s status. In the proposed method, a penalty-and-reward function is designed. If a user types as they should, then they earn a reward, leading to an increase in the trust level. However, if the typing of the user does not comply with the template, a penalty is given, causing the trust level to decrease and the system’s confidence in the user’s authenticity to decrease. The user will be locked out of the system if the trust level is lower than a threshold. Mondal and Bours [69] designed a continuous authentication system using mouse dynamics. In this work, the authors employed a public mouse dynamics dataset containing 49 users together with six machine learning algorithms (e.g., support vector machine and decision tree learning) to evaluate the proposed system. Temper et al. [70] developed a fuzzy classifier-based continuous authentication system aiming to secure mobile devices. The proposed system uses behavioral biometric traits (e.g., touchscreen gestures) as unique features for authentication. Experimental results show that the proposed system achieves an EER of 11.5% on a private dataset, collected from 22 different users. Traoré et al. [71] presented a continuous authentication framework to address the threat of cheating in online tests. In the proposed framework, three biometric traits, namely face, mouse dynamics and keystroke dynamics, are collected and processed during the exam. Evaluated with offline datasets, the proposed framework exhibits encouraging results.

#### 4.1.5. Biometric Authentication versus Password- and Token-Based Authentication

For a long time, password-based authentication (according to something you know) has been the standard technique for authentication in the IoT environment due to its simplicity and convenience. The fundamental problem with passwords can be explained succinctly—a short and memorable password can be easily guessed, while a long and complex password is difficult to remember [72]. Token-based authentication (according to something you have) performs authentication with a token, which is a secure storage device that contains passwords or generates one-time passcodes [72]. Unlike traditional password- and token-based authentication, biometric authentication uses features from human biometric traits (e.g., fingerprint, face, and iris) for authentication purposes. A biometric trait is unique to its owner; thus, it cannot be shared or stolen, an attribute passwords and tokens do not have [73]. Moreover, biometrics have the property of nonrepudiation. This property ensures that a user participating in a transaction cannot subsequently reject it as unauthorized or claim not to have participated in the transaction [72].

The performance of a biometric authentication system in the IoT is usually evaluated by metrics such as the FAR and FRR [74], as discussed in Section 3.2. If the performance of password- and token-based authentication systems is represented in a similar fashion to biometric-based authentication systems using those metrics, then for password-based authentication systems, the FAR corresponds to the percentage of successfully guessing the password and the FRR means the percentage of accidental mistakes made by users at input, while for token-based authentication systems, the FAR and FRR indicate the chances of a token being stolen or lost due to ownership factors [75]. In password- and token-based authentication, users may blame themselves for authentication failure. However, for biometric-based authentication systems, because of biometric uncertainty, although a user is not at any fault, the system can still reject the user [72]. As shown in Table 2, adoption of discriminative biometrics (e.g., using iris rather than voice) or multi-modal biometric systems may improve authentication performance. However, factors such as user convenience, acceptability, and resource constraints of IoT devices should be considered in the design of biometric authentication systems in the IoT.

### 4.2. Biometric Authentication and Key Agreement

As authentication and key agreement are two of the main security requirements in IoT applications [2], several existing research articles discuss both authentication and key agreement. Xie et al. [76] proposed a smart card- and password-based two-factor user authentication scheme for mutual authentication and key management between the user and server. The proposed scheme can defend several common attacks (e.g., smart card stolen attacks and password guessing). Improving on [56], Lu et al. [77] developed anonymous authentication and key exchange in the mobile client-server environment. The proposed scheme is immune to attacks such as user impersonation, insider, and trace attacks, which the scheme in [56] suffers.

To further strengthen the security of two-factor user authentication and key agreement, biometrics is utilized as the third factor to withstand the stolen card attacks. For example, Yoon and Yoo [78] combined biometric-based authentication with key agreement for secure authentication in multi-server communication environments. A reinforced user authentication function is obtained by using biometrics, while a strong key agreement is provided by adopting the elliptic curve cryptosystem, reducing the computational load on smart cards. Wazid et al. [79] proposed secure biometric-based user authentication and key agreement for cloud computing with additional functionalities, such as efficient password and biometric data update and support for multi-server environments.

*Biometric authentication and key agreement with privacy protection*: Although incorporating biometrics can enhance the security level of the key agreement process between the user and server, vulnerabilities associated with biometric data in the key agreement process may lead to exposure of user identity and privacy. To resolve this issue, Chuang and Chen [80] presented a multi-server authentication and key agreement method using three factors, smart cards, password and biometrics to ensure user anonymity and defend several types of attacks. This method is claimed to be lightweight and cost-effective. Mishra et al. [81] improved Chuang and Chen’s method in [80], claiming that it is vulnerable to the stolen smart card attack and the denial-of-service attack. The improved method can prevent adversaries from obtaining the previously established session keys from the stolen smart card, while keeping the merits of Chuang and Chen’s method (e.g., user anonymity and low computational cost). Zhang et al. [82] designed a privacy protection scheme, which can provide biometric authentication at the server side and make the biometric template unknown to the server in the e-health environment. In this way, user privacy is preserved in the authentication and key agreement process.

*Group-based biometric authentication and key agreement*: Group-based authentication and key agreement is a promising technique to tackle issues such as congestion and overburden. Kakarla and Singamsetty [83] proposed a lightweight group-based authentication and key agreement protocol using elliptic-curve Diffie–Hellman cryptography. With this protocol, the server can authenticate devices in the group simultaneously in wireless networks, while utilizing bandwidth efficiently by reducing congestions. Cryptanalysis and performance evaluation of the proposed protocol show favorable outcomes. Modiri et al. [84] introduced a group-based lightweight authentication and key agreement to provide mutual authentication for a large number of heterogeneous mobile devices. Because the proposed scheme involves only hash functions rather than symmetric or asymmetric encryption operations, it achieves considerably better performance than the existing methods in terms of network overheads. The use of biometrics in group-based authentication and key agreement heightens the security level. Adhikari et al. [85] proposed a group-based authentication and key agreement protocol, which creates two groups among devices based on the device type and uses biometric authentication to enhance network security. Security analysis substantiates the security requirements (e.g., preservation, confidentiality, and mutual authentication) attained by the proposed protocol.

In this sub-section, several authentication and key agreement schemes are reviewed, in which biometrics are used as one factor to enhance the security level of the overall system. Despite the benefits of biometrics (e.g., difficult to copy or share, cannot be lost or forgotten and hard to forge, as opposed to passwords [62]), biometric data are uniquely linked to users’ identity. Therefore, user privacy protection is becoming more important, calling for attention and action from both academia and industry. In addition, according to a survey conducted by Chuang and Lei [86], most existing mutual authentication and key agreement schemes cannot simultaneously meet all the requirements, such as security, user anonymity, public key management, and independent authentication. Therefore, more effort is required to design better biometric authentication and key agreement systems.

## 5. Biometric-Cryptographic Systems for IoT Data Encryption/Decryption

**Figure 4:**
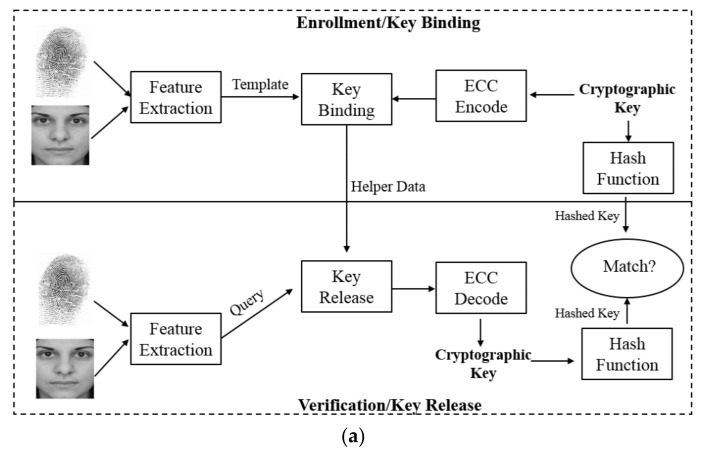

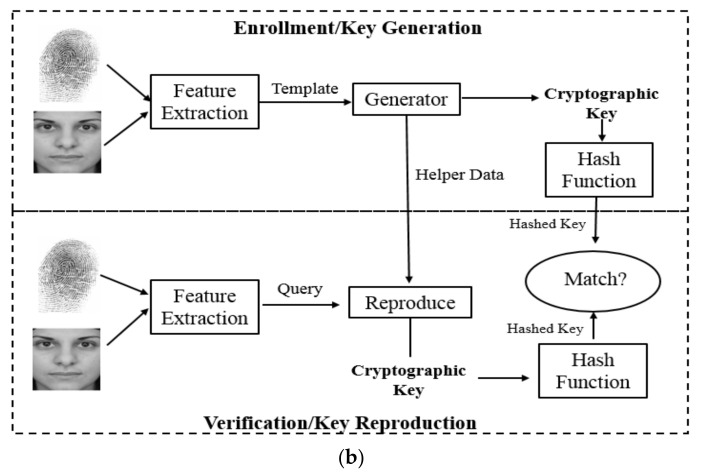
The enrollment and verification processes of (**a**) biometric key binding systems, and (**b**) biometric key generation systems (adapted from [93]). In the figure, ECC means error correction code.

Encryption ensures that sensitive data collected by IoT devices are protected and unaltered during transmission between IoT devices and the server. Encryption is usually based on cryptography in which secret keys are used in the data encryption and decryption processes. With data encryption, security threats, such as eavesdropping, can be prevented. However, all the biometric-based authentication systems discussed in Section 4 can only output a binary decision, either acceptance or rejection, without functions of data encryption and decryption [75]. Equipped with these functions is a technique called biometric-cryptography, or bio-cryptosystem in short, a combination of biometrics and cryptography and taking advantage of both. As the key in a bio-cryptosystem for IoT data encoding and decoding is the same, the encryption and decryption algorithms are symmetric. Specifically, in a biometric-cryptographic system, a secret key can be seamlessly bound with biometric data using a fuzzy commitment or fuzzy vault, of which the enrollment and verification processes are shown in Figure 4a.

The fuzzy commitment [87] takes an original binary template feature vector (e.g., B_T_) as input. During the enrollment/encoding phase, a secret key (e.g., k) is encoded by the BCH encoding scheme to produce a codeword (e.g., C). The codeword C and the original template data B_T_ are merged by the XOR operation to generate E, that is, E = B_T_ ⊕ C. The hash value of k (e.g., hash(k)) is calculated and stored together with E in the database. In the verification/decoding phase, the query feature vector (e.g., B_Q_) in the binary format is XORed with E to obtain C1 = B_Q_ ⊕ E. A BCH decoding algorithm is applied to C1 to obtain k1. Then, the hash value of k1 (e.g., hash(k1)) is computed and compared with hash(k). If hash(k1) = hash(k), then k1 = k, which means that the secret key k is successfully retrieved.

The fuzzy vault [88] takes an unordered template feature set (e.g., B_T_) as input. Here “unordered” means that the position of an element in the feature set does not alter the characteristics of the features. For example, the set {1, 2, 3} carries the same information as {3, 1, 2}. During the enrollment/encoding phase, the secret key (e.g., k) is encoded into a vault and locked by the template feature set B_T_ through a polynomial P of variable x. The segments of k are used as the coefficients of P. We first obtain the polynomial projections P(B_T_) for the elements of B_T_, and then randomly generate chaff points that do not lie on P to arrive at the final point set R. In the verification/decoding phase, only when the query feature set (e.g., B_Q_) overlaps with B_T_ to a large extent can the vault be unlocked to retrieve the secret key k. In other words, if many points in R that lie on P can be located, the polynomial P can be reconstructed, and thus the secret key k can be retrieved.

Conversely, a secret key can be directly generated from the biometric data using a fuzzy extractor, of which the enrollment and verification processes are shown in Figure 4b. In this section, several recent schemes in the literature using the biometric-cryptographic technique are summarized and discussed below.

Karimian et al. [36] proposed an ECG-based bio-cryptosystem for IoT devices, given that ECG signals can be easily acquired using suitable IoT devices, and it is extremely hard to steal and spoof a user’s ECG signal. Zheng et al. [89] investigated the security of the ECG-based bio-cryptosystem for wearable and implantable medical devices. The authors conducted a detailed analysis on two cryptographic primitives, the fuzzy commitment and fuzzy vault, and discussed their strengths and weaknesses when used in ECG-based bio-cryptosystems. Choi et al. [90] protected the cryptographic key stored in the unmanned IoT device with a novel two-factor fuzzy commitment. The proposed scheme uses both the biometric data and physical unclonable function (PUF), which increases the difficulty for the attacker to acquire the correct key.

Wazid et al. [91] devised an authentication mechanism called LAM-CIoT in the cloud-based IoT environment. Through LAM-CIoT, the user can access the IoT data remotely in a secure way. In addition, a fuzzy extractor is employed at the user side for biometric-based local verification such that the user’s biometric data can be protected. Performance analysis demonstrates that the proposed method has better security and lower computational overhead than similar schemes. Ebrahimi and Sarmadi et al. [92] designed an enhanced fuzzy extractor using hardware and software, which can extract secure and reproducible keys from biometric data and can be applied to resource-constrained IoT devices.

## 6. Present Challenges

Because of its massive scalability and coverage, the study of IoT has attracted many researchers. Over the past decade, much has been done in different areas of IoT, including application development, security, privacy protection and connectivity. However, IoT is still in the development stage and more work is required to improve its functionality. Research challenges are present, such as device power consumption, battery limitation, memory storage space, cost of performance, security, and convenience, all of which call for action in the further study of IoT. This section identifies and highlights challenges that need addressing to ensure future success in applying biometrics to the IoT domain.

### 6.1. Vulnerabilities of Biometric Systems and Their Impact on IoT Security

**Figure 5:**
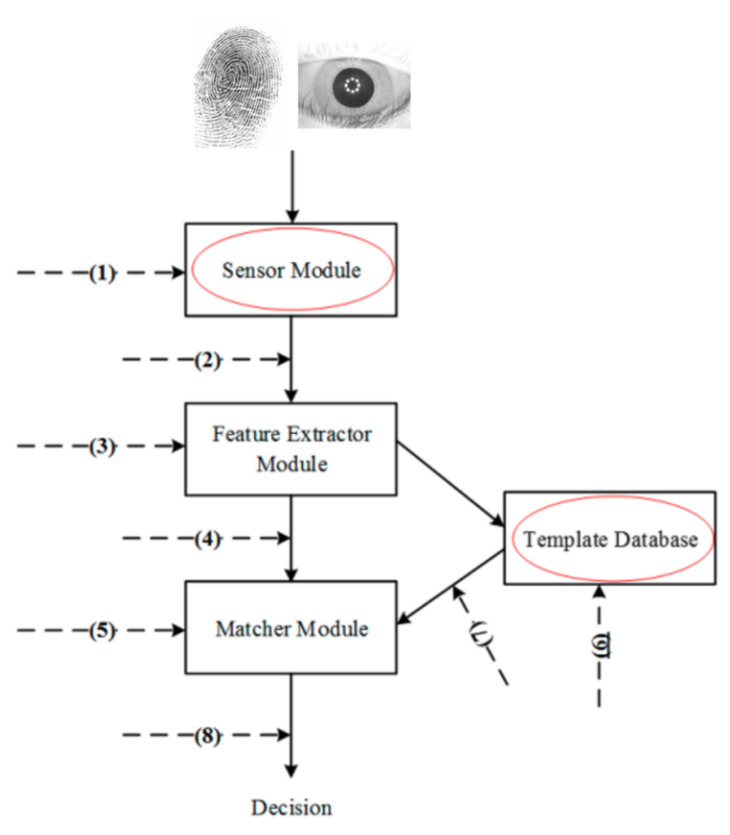
Eight possible attack points targeting biometric authentication systems. Two of them highlighted in the red circle are discussed in this work (adapted from [22]).

The implementation of biometric systems in the IoT can hardly avoid security vulnerabilities that come with biometric authentication itself. The situation may be aggravated by the inherent characteristics of the IoT, such as limited resources and scalability, making biometric systems more vulnerable to certain attacks. There are various attacks targeting biometric authentication systems and Ratha et al. [94] summarized eight different points of attacks, as shown in Figure 5. Among them, the attacks (e.g., spoofing attacks) to user interface at point (1) and the attacks (e.g., stealing biometric templates) to the biometric template database at point (6) are possibly the two most serious threats to IoT users’ privacy [22] and therefore discussed in detail in this work.

At point (1), spoofing attacks to the user interface with fake biometric traits are common because biometric traits (e.g., face, fingerprint) are not secret, and adversaries can obtain them and use forged traits to spoof biometric systems. In the IoT environment, many IoT devices are unmanned and not equipped with liveness detection. For instance, the Touch ID of an iPhone can be fooled by a fake fingerprint film made from glue [95]. Since multi-modal biometric authentication systems are more robust than their unimodal counterparts [96], incorporating multi-modal biometrics can mitigate the threat of spoofing attacks because it is more difficult to spoof two or more modalities than a single one [97]. However, multi-modal biometric systems are not the ideal remedy for spoofing attacks given the resource limitation of IoT devices. Using the biometric traits possessing the liveness property (e.g., ECG) can be a better option [89], but it still depends on specific IoT applications because, in most applications, sensors may not be equipped with the ECG collection function.

At point (6), attacks such as stealing or modifying biometric templates have severe consequences on users’ privacy. This is because biometric template data are uniquely linked to users’ identity and biometric traits cannot be revoked or reset similar to passwords or tokens. In the IoT context, with a large amount of sensitive data (including biometric data) collected and stored on IoT devices or servers, the privacy concern is more critical and security requirements are more demanding. Moreover, the IoT architecture is intended for automation without human intervention, making the task of protecting sensitive and important information (e.g., biometric templates) more challenging [98].

### 6.2. Selection of Biometric Traits for IoT-oriented Authentication

As discussed in Section 4.1, each biometric trait has its own characteristics, leading to vastly different authentication performance. Certain biometric traits (e.g., iris) contain more discriminative features and therefore enable better authentication accuracy than other biometric traits (e.g., voice) [4]. However, IoT applications are diverse, and there is no single biometric trait that can meet all the requirements of IoT-oriented authentication scenarios. For example, despite the strong authentication performance offered by using iris, voice is clearly a better choice than iris in the authentication scenario of smart speakers. Similarly, using multi-modal biometrics enhances authentication accuracy and security, but multi-modal biometric systems have implications of making the overall system more complicated and cost-ineffective, thereby increasing the storage, processing, and computational burden of IoT devices.

It follows from the above analysis that selection of biometric traits and whether to use a multi-modal biometric system should be carefully considered and based on specific IoT applications. Any injudicious choice of single-modal or multi-modal biometrics not only causes user inconvenience, but also increases resource consumption in the IoT. Unfortunately, to date, there have been no guidelines on the selection of appropriate biometric traits in the implementation of biometric systems for IoT security.

### 6.3. Limitations of Applying Biometric-Cryptographic Techniques to IoT Data Encryption/Decryption

In Section 5, several biometric-cryptography-based schemes, such as fuzzy commitment, fuzzy vault and fuzzy extractor, are introduced and discussed. In the design of biometric authentication systems for the IoT, biometric-cryptographic techniques are not a common option for two reasons. The first reason is that cryptographic key binding or generation involves a large amount of computation, thus imposing heavy computing costs on resource-constrained IoT devices [99]. The second reason is that often the currently available biometric-cryptographic techniques (e.g., fuzzy commitment and fuzzy vault) cannot be directly applied to the feature sets formed in the unencrypted domain. This is because these techniques require fixed-length feature representations [100] in order to measure feature disparity using metrics such as hamming distance or set difference, but converting raw biometric data collected by IoT devices to the required formats not only creates extra computational load, but also likely decreases authentication accuracy.

### 6.4. Uncertainty of Biometric Data

Biometric data contain many uncertainties such as intraclass variability and interclass similarity. Using the most common biometric authentication, fingerprint recognition, as an example, when a contact sensor is used to capture live finger images, nonlinear distortion and rotation of fingerprints are inevitable due to skin elasticity, skin moisture content, finger displacement, contact pressure, sensor noise and imaging methodology [101]. Because of the uncertainty in the captured fingerprint data, matching between query and template fingerprints could fail. Therefore, biometric authentication is inherently a probabilistic task and there is inevitable uncertainty and the risk of error, although the technology and the system itself behave as designed. Despite these difficulties, there is ongoing research to improve the quality and discriminative power of biometric data as well as the matching performance of biometric authentication systems, such that their role in safeguarding IoT security is more effective.

### 6.5. Limited Resources of IoT Devices

Many IoT devices have limited computing resources [11]. Biometric recognition in an IoT setting, which includes complex calculations such as data processing, matching and decision making, can incur more costs and add a heavier burden to IoT devices than traditional password-based authentication. Therefore, any new biometric authentication systems should optimize the usage of limited storage and battery power of IoT devices while responding swiftly to authentication requests [11]. Moreover, the implementation of a biometric system directly relies on the hardware of an IoT device. For example, IoT devices without camera sensors cannot have face-based authentication.

## 7. Potential Solutions and Opportunities

To address the above challenges brought about by applying biometric authentication to the IoT, researchers have made remarkable efforts in proposing different strategies and solutions to certain challenges, which are analyzed and discussed below.

### 7.1. Protecting Biometric Template Data

Biometric systems provide authentication between users and IoT devices [102], however, during the authentication process, users’ sensitive biometric data should be protected. The anonymous technology can be utilized to handle user-identifiable information, but we need to balance the efficacy of privacy protection with the accuracy of results [102]. To achieve this goal, privacy-preserving techniques, such as cancelable biometrics [103] and homomorphic encryption [104], are effective methods in the latest research outcomes. For cancelable biometrics, in the enrollment phase, instead of storing the original biometric data, they are transformed by a non-invertible transformation function to achieve template data protection. In the verification phase, the same non-invertible transform is applied to the query data. Matching between the template and query data is conducted in the transformed domain to reduce the risk of biometric data leakage [105]. Homomorphic encryption is a technique that enables mathematic operations on encrypted data without the involvement of the decryption key. This allows the confidentiality of biometric data to be protected and matching to be performed between encrypted template data and encrypted query data without degrading recognition accuracy. As long as the decryption key is safe, the system is cryptographically secure [106].

Cancelable biometric authentication aimed for IoT security is proposed in the following research papers. Yang et al. [4] developed a biometric authentication system for IoT devices based on both iris recognition and steganography techniques. In the proposed approach, system security is increased by using random projection-based cancelable biometrics and steganography to hide the user-specific key in cancelable template generation. Punithavathi et al. [107] introduced a cloud-based lightweight cancelable biometric authentication system, which protects template data with the random projection-based cancelable biometric technique. The random projection transforms data points into another version using random orthonormal matrices. In the meantime, distances between data points before and after the transformation are preserved. Punithavathi and Geetha [108] provided a partial DCT (Discrete Cosine Transformation)-based privacy-preserving cancelable biometric authentication framework applicable to the IoT. The proposed framework also comprises a session key agreement and data encryption.

In addition to cancelable biometrics, homomorphic encryption is another technique that renders privacy preservation while maintaining recognition accuracy. Farid et al. [109] proposed an identity management framework for IoT and cloud computing-based healthcare systems using ECG and photoplethysmogram (PPG) signals. To protect users’ sensitive biometric data, homomorphic encryption is performed such that data processing and analysis can be conducted in the encrypted domain in the cloud. The proposed framework is evaluated using a machine learning model on a dataset of 25 users. The experimental results show significant improvements in overall performance in terms of authentication accuracy and security, compared to that using just ECG or PPG signals. Although homomorphic encryption applied to biometric authentication enables matching of biometric data in the encrypted domain and generates same matching results as those obtained in the unencrypted domain, how to reduce algorithm complexity and improve computational efficiency is an open question. More work is required in this direction to further cut down computational cost [104].

### 7.2. Reducing the Impact of Biometric Uncertainty

Biometric uncertainty, produced at biometric image acquisition, can degrade the performance of biometric authentication systems in terms of recognition accuracy. To mitigate the negative impact of biometric uncertainty and achieve satisfactory matching performance, apart from multi-modal biometrics discussed in Section 4.1, techniques such as discriminative feature representation and deep/machine learning can act as countermeasures to biometric uncertainty.

One way of reducing the adverse effect of biometric uncertainty is to use stable and discriminative feature representation. For example, Zheng et al. [110] applied finger-to-heart biometric authentication to implantable medical IoT devices. In the proposed system, fingerprints are used to perform authentication for access control over implantable medical IoT devices. To achieve good recognition accuracy, the authors adopted and improved the well-known fingerprint minutia descriptor, Minutia Cylinder-Code (MCC). Only a subset of MCC feature data is chosen to save the limited storage and computing resources of implantable medical IoT devices. Due to MCC’s strong discriminative power and stability in combating biometric uncertainty, the proposed system achieves satisfactory recognition accuracy, albeit with only a subset of MCC feature data.

Another way of tackling biometric uncertainty to attempt good matching performance is the employment of powerful deep learning techniques (e.g., CNN). For example, to decrease noise in ECG signals and improve recognition accuracy, Zhang [111] adopted the deep learning technique and proposed a CNN-enabled ECG-based biometric identification framework for IoT applications. In this scheme, features from raw ECG data are learned directly by the CNN without the need of manual feature extraction. The experimental results on the MIT-BIH normal sinus rhythm database show that the system obtains an identification rate of 99%, outperforming other state-of-the-art methods. Yang et al. [112] developed a deep learning-based privacy-preserving finger-vein authentication system for IoT edge devices. By employing the binary decision diagram, the designed template protection algorithm is resource-saving and secures the finger-vein template data, while achieving competitive recognition performance.

### 7.3. Lightweight Algorithm Design

Since it is necessary to process a large amount of data generated by a huge number of interconnected devices in IoT, energy consumption is substantial, affecting the battery life of IoT devices. Therefore, lightweight and green mechanisms have been proposed for IoT devices to make biometric systems more energy efficient. For example, Yang et al. [30] designed a lightweight fingerprint recognition system for securing IoT devices. By applying an efficient XOR operation to fingerprint feature vectors, the authors reduced the size of resultant templates. Meanwhile, the proposed system has an unknown ‘key’ (i.e., the feature data itself), which is discarded afterwards and not stored anywhere. Dhillon and Kalra [113] presented a lightweight biometric system for remote user authentication of IoT devices. It is shown in the paper that the proposed scheme is robust against multiple attacks such as eavesdropping, man-in-the-middle and denial of service attacks. Taher et al. [114] worked out a lightweight and secure mutual authentication protocol that satisfies the constraints of IoT devices, such as limited power and computing capability. The proposed protocol has a three-level feature extractor to extract features from biometric images. Moreover, a one-way hash function and XOR operations are employed in order to ward off various malicious attacks and realize efficient computations.

### 7.4. Biometrics with Other Technologies

Biometrics for IoT applications can overcome drawbacks of traditional password-based authentication and thus enhance the security of IoT networks; however, biometrics alone cannot be the cure to all the security issues of the IoT. The use of biometrics together with other technologies (e.g., machine learning, blockchain, and edge computing) can bring more benefits to IoT security [115]. For example, machine learning is considered a powerful tool. Research works, such as [34,41,69], apply machine learning to improve system performance or reduce biometric uncertainty. Blockchain, referred to as a decentralized ledger maintained by a number of independent users, is an emerging technology to protect transactions against forgery by adding a digital signature [116]. In a system that uses both blockchain and the IoT to generate blockchain transactions, the verification of a reliable creator is a challenge, but the use of biometrics can ensure that the creator of a transaction is the correct owner of the private key [117]. As an extension of cloud computing, edge computing has experienced fast development. The use of edge computing in the IoT may solve issues of cloud computing (e.g., processing of a huge amount of data and/or a long distance between servers and users) by placing small edge servers between the users and the cloud. In this way, the communication cost is low, and data are safer without being transmitted to the remote cloud server. It has been proven that using biometric authentication in edge computing-based IoT applications can improve system security [118]. It will further strengthen the security of IoT devices and the sensitive information therein if the privacy-preserving technique can be applied to protect biometric data themselves.

## 8. Threats to the Validity of This Survey

It is acknowledged that the main threats to the validity of this survey are possible neglect in the selection of research articles [119] and imperfection in classification and summarization.

To manage the selection of research articles, we define the research questions and scopes in advance and make the article selection a carefully planned multiple-step task, including the design of inclusion and exclusion criteria for filtering purposes. We also adopt a variety of search engines to check the completeness of selected articles. However, given that it is a non-trivial task, it is difficult to detect and include all relevant research articles in this survey without missing any important research work.

To make classification and summarization as appropriate and accurate as possible, we perform the classification of research articles based on titles, keywords, and abstracts with cross-checking among the authors. Classification is a challenging task due to the lack of a standard framework for classifying different studies in biometrics and the IoT. At the end of each section, we give a summary. There may be imperfection in the summarization in that it heavily relies on the authors’ knowledge and research experience.

## 9. Conclusions

This review paper scrutinizes a range of biometric systems or techniques for addressing vulnerabilities of different layers of the IoT architecture. Attention is paid to the authentication and encryption aspects of biometric-based systems for the sake of IoT security. Regarding authentication, contemporary biometric systems are discussed and categorized into single-modal or multi-modal biometric authentication systems based on the types and number of biometric traits used. Regarding encryption, bio-cryptosystems utilizing different cryptographic techniques are reviewed. We emphasize that each biometric trait or a combination of traits has strengths as well as noteworthy shortcomings. Moreover, despite the significant advantages of applying biometrics to safeguarding IoT security, potential challenges are highlighted, and possible solutions are presented. To overcome the challenges identified, the authors suggest the following future research directions:

- Because no single biometric trait can satisfy the needs of all IoT applications, how to select suitable biometric traits for IoT-oriented biometric authentication is a nontrivial task, calling for more research attention. Moreover, although multi-modal biometric systems can reduce the effect of biometric uncertainty and bring about higher authentication accuracy than single-modal biometric systems, the extra cost incurred (e.g., additional processing and computing time) should be taken into consideration. Due to the resource limitations of IoT devices, how to design efficient and cost-effective multi-modal biometric systems is a much-needed research topic. For example, capturing iris and face biometrics simultaneously saves data collection time and brings convenience to users;
- The implementation of biometric-cryptographic techniques (e.g., fuzzy vault and fuzzy commitment) can provide both authentication and data encryption/decryption for IoT, but the large computing cost of the cryptographic key binding or generation operation and possible performance degradation are certain drawbacks of bio-cryptosystems. Therefore, more research effort should be directed to the development of new biometric-cryptographic techniques which can save cost, while providing satisfactory authentication performance in the IoT environment;
- The spoofing attack to the user interface is a serious security issue concerning biometric systems in the IoT, and the situation is made worse due to IoT’s automatization requirement. To the best of our knowledge thus far, there has been little study on this issue in the IoT field; thus, urgent research activities are required to defy spoofing attacks to IoT devices, especially in IoT applications with no human intervention;
- Despite its importance, biometrics for IoT security is a relatively new research area, evidenced by the limited number of articles that can be found in the literature. Given the resource constraints of IoT devices and the issue of user acceptability and/or convenience of collecting biometrics, it is necessary to develop lightweight authentication schemes, preferably with functions such as template data protection or key management so as to strengthen system security. Moreover, a user-friendly course of action is another critical factor to encourage public acceptance of biometrics in the IoT. We believe that the key to the widespread deployment of biometric systems in the IoT is to strike the right balance between privacy and convenience.

## Figures and Tables

**Table 1:** Attacks on different layers and security requirements [2].

Layers	Attacks	Description	Security Requirements
**Perception Layer**	Node Tampering	The attacker physically alters the node to obtain sensitive information.	Authentication, Data Confidentiality, Lightweight Encryption, Key Agreement
	RF Interference	The attacker sends noise signals in the radio frequency spectrum.	
	Node Jamming	The attacker disturbs the wireless communication using jammers.	
	Malicious Node Injection	The attacker injects malicious nodes in the network, which can modify data and pass wrong data to other nodes.	
	Physical Damage	The attacker physically harms components of IoT.	
	Malicious Code Injection	The attacker introduces malicious code into the nodes of IoT to obtain control of the IoT system.	
**Network Layer**	Traffic Analysis Attacks	The attacker intercepts and examines messages to obtain network data.	Authentication, Key Management, Intrusion Detection, Communication Security, Routing Security
	RFID Spoofing and Cloning	The attacker spoofs RFID signals, copies data from a pre-existing RFID tag to another RFID tag.	
	Man-in-the-middle Attacks	The attacker intercepts the communication between two nodes in the wireless channel.	
	Routing Information Attacks	The attacker spoofs, modifies or sends wrong routing information to complicate the network.	
	Denial of Service	The attacker creates a large amount of traffic to flood the network such that the intended users cannot access services.	
	Sybil Attacks	The malicious node takes the identifies of multiple nodes and acts as them.	
**Application Layer**	Phishing Attacks	The attacker obtains private information through spoofing.	Authentication, Information Security Management, Privacy protection
	Viruses, Worms, Trojan Horses	The attacker undermines the system using malicious code such as viruses, worms and trojan horses.	
	Denial of Service	The attacker blocks users from the application layer by denying services.	

**Table 2:** The biometric methods for IoT security and corresponding information.

Method for IoT Security	Year of Publication	Type of Biometric Traits	Databases	Performance	Hardware/Platform
**Single-Modal Biometrics**					
Devikar et al. [25]	2016	Fingerprint	-	-	ESP8266 NodeMCU
Shah and Bharadi [26]	2016	Fingerprint	-	-	Raspberry Pi
Prakash and Venkatram [27]	2016	Fingerprint	-	-	Raspberry Pi 2
Taheri and Yuan [28]	2018	Fingerprint	-	-	Simulator on PC
Sarika et al. [29]	2019	Fingerprint	-	-	Arduino board
Yang et al. [30]	2019	Fingerprint	FVC2002DB3	EER = 3%	Simulator
Golec et al. [31]	2020	Fingerprint	Private	EER = 30%	Raspberry Pi
Hossain et al. [32]	2016	Face	FERET	RA = 99.5%	Simulator
Thilagavathi and Suthendran [33]	2018	Face	-	-	Simulator on Web Server (Apache)
Gayathri et al. [34]	2020	Face	-	-	Simulator on PC
Kolhar et al. [35]	2020	Face	WIDER FACE	RA = 60.7%	Raspberry Pi
Karimian et al. [36]	2016	ECG	-	-	-
Hussein et al. [37]	2017	ECG	MIT-BIH	RA = 97.78%	Raspberry Pi 3
Barros et al. [38]	2019	ECG	NIH PhysioBank	RA = 98.2%	Simulator on PC
Karimian et al. [39]	2019	ECG	PTB	RA = 98.76%	Simulator
Shin and Jun [40]	2015	Voice	-	-	-
Duraibi et al. [41]	2020	Voice	-	-	-
Lu et al. [42]	2017	Finger-vein	MMCBNU_6000	EER = 0.36%	Simulator
Yang et al. [4]	2019	Iris	CASIA-Iris v.3	EER = 0.22%	Simulator
Gad et al. [43]	2019	Iris	CASIA-Iris v.4	EER = 0.20%	Simulator
**Multi-Modal Biometrics**					
Macek et al. [44]	2016	Face & Iris	CASIA-Face v.5 CASIA-Iris v.4	RA = 99.1%	Simulator
Shahim et al. [45]	2016	Hand & Gesture	-	-	-
Olazabal et al. [46]	2019	Face & Voice	CSUF-SG5	Fused: EER = 8.04% Face: EER = 14.05% Voice: EER = 43.76%	Raspberry Pi 3 Model B
Hassen et al. [47]	2020	Fingerprint & Finger-vein	-	-	Simulator on PC
Cherifi et al. [48]	2021	Ear & Arm Gestures	AWE HMOG	Fused: EER = 5.15% Ear: EER = 20.80% Arm: EER = 10.60%	Simulator
